# Weighted Blankets and Sleep Quality in Children with Autism Spectrum Disorders: A Single-Subject Design

**DOI:** 10.3390/children8010010

**Published:** 2020-12-27

**Authors:** Bryan M. Gee, Kimberly Lloyd, Jesse Sutton, Tyler McOmber

**Affiliations:** 1 College of Rehabilitation Sciences, Rocky Mountain University of Health Professions, Provo, UT 84606, USA; 2 School of Rehabilitation and Communication Sciences, Idaho State University, Pocatello, ID 83209, USA; lloykim3@isu.edu; 3 Mary Lanning Healthcare, Hastings, NE 68901, USA; jessesutton.38@gmail.com; 4 Idaho Home Health and Hospice, Gooding, ID 83330, USA; tcmcomber@gmail.com

**Keywords:** weighted blankets, sensory-based interventions, autism spectrum disorder

## Abstract

The purpose of the study was to explore the efficacy of weighted blanket applications and sleep quality in children with autism spectrum disorder and behavioral manifestations of sensory processing deficits. Two 4-year-old participants diagnosed with autism spectrum disorder who also experienced sleep disturbances took part in a single-subject design study. Objective sleep measures and caregiver surveys were tracked for a baseline period of eight days, followed by a 14-day weighted blanket intervention and a seven-day withdrawal phase. Caregiver reports and objective data were evaluated using visual analysis and the percentage of non-overlapping data methods. The results suggest minimal changes in sleep patterns as a result of the weighted blanket intervention. The findings based on using a weighted blanket intervention were enhanced morning mood after night use and a significantly decreased time to fall asleep for participants, though they were not strong enough to recommend for clinical use. Future directions include single-subject and cohort-designed studies exploring the efficacy of weighted blankets with increasing sleep quality among children with autism.

## 1. Introduction

Autism is a complex neurodevelopmental condition with hallmark features that include atypical language and communication skills, poor social interaction, impaired executive functioning, sensory processing, and motor skill coordination [1]. The condition presents with comorbid psychiatric and medical conditions which may include anxiety disorder, oppositional defiant disorder, attention-deficit/hyperactivity disorder, intellectual disability, immune system irregularities, gastrointestinal disorder, sleep disturbances and epilepsy and seizure disorder [2]. As mentioned with the medical conditions, are sleep disturbances and low sleep quality [3] is the focus of this paper. Research estimates that 44% to 83% of individuals (adults and children) with autism experience sleep disturbances [4]. Humphreys et al. [5] reported a reduced sleep duration of 17 to 43 min in children (30 months to 11 years) with autism as compared to children without autism.

Additionally, children (18 to 42 months) with autism have impaired sleep patterns [5]. Sleep duration are shortened in children with autism due to later bedtimes, earlier risings, and frequent wakings (three or more wakings a night) [5]. Malow et al. [6] found that those children with autism who slept poorly showed a decrease in rapid eye movement (REM) sleep and an increase in non-rapid eye movement (NREM) sleep stage 4. Malow et al. [6] also reported that children with autism who have a sleep disorder show an exacerbation of behavioral challenges throughout the next day. Children with autism can have sleep impairments, which can adversely affect their quality of life with an increase in aggressive behavior, anxiety, increased parental stress, and family life quality [7].

Further confounding the issue is the prevalence of sensory processing disturbances among children with autism has upon activities of daily living, particularly sleep. A systematic review conducted by Ismael et al. [8] reported that the majority children with autism experience sensory disturbances that impact sleep, primarily in the domain of sensory avoiding or sensory over responsivity. In a study using behavioral and physiological measures, Reynolds et al. [9] found that children aged six to 12 years with autism have a higher prevalence of atypical sensory behaviors (sensory under responsiveness and over responsiveness) and sleep disturbances than typical children of the same age.

Commonly used interventions to support sleep quality among children with autism include pharmacological agents [10], behavioral and contextual sleep hygiene changes [8], caregiver education, and training [11]. However, an intervention that has seen increased use in attempting to improve sleep quality among children with autism is a weighted blanket [12].

Weighted blankets are used as an intervention strategy to improve sleep in children with autism who have sleep impairments [12,13]. The current underlying posit for weighted blanket use is to provide deep touch pressure stimuli, thus acting as a calming mediator by increasing parasympathetic activity [13,14]. The mediating intervention in the blanket is weight imbedded into the blanket primarily through plastic beads or balls to approximately 10% of the user’s body weight [12,13,14,15,16]. The weight is either modified through pouches and pockets with interchangeable weights or are more permanent with non-modifiable weight. Weighted blankets are passive sensory-based adjunctive intervention that is applied to a child or adult to reduce unwanted behaviors rooted in sensory modulation impairments [12,13]. Some authors state that weighted blankets help individuals stabilize and modulate sensory input and lower anxiety during stressful situations by enhancing parasympathetic activation [17,18].

Reviewing the literature related to sensory processing and children without a neurological or behavioral impairment (including children with autism) yielded some sparse resources. Foitzik and Brown [19] reported that typically developing school-aged children who demonstrate sensory disturbances with tactile sensory processing (hyporesponsive) slept longer and had fewer night wakings. Furthermore, Fiotzik and Brown [19] reported that children and adults demonstrate fewer sleep quality disturbances in conjunction with more typical sensory processing patterns than those with sensory processing related behaviors [19]. Vriend et al. [20] reported that children with low sleep quality may demonstrate difficulty in sensory processing domains, specifically emotional regulation. In a study of sensory processing and sleep among infants and toddlers, Vasak et al. [21] reported correlations between increased sensory seeking behaviors and shorter daytime sleep duration (naps). Such correlations also applied to increased sensory sensitivity behaviors and increased time to fall asleep (at night). In school-aged children diagnosed with attention deficit hyperactivity disorder (ADHD), Shochat, Tzischinsky, and Engel-Yeager [22] reported that children with ADHD experienced disturbances with sleep quality in part due to tactile sensory over-responsivity (SOR).

While sleep disturbances are commonplace in children with autism, minimal empirical evidence exists examining potential interventions to enhance sleep quality using sensory-based interventions [12]. Parents and caregivers often seek strategies to increase sleep quality and duration for their children with autism [12]. Some of the literature in occupational therapy has described applying sensory-based intervention to influence a child’s level of arousal, behavioral organization, and on-task behavior [15]. One potential sensory-based strategy to enhance sleep patterns in children with autism is the use of a weighted blanket [15].

Sensory integration theory [22,23] posits that deep pressure sensory stimulation (touch) may create calming effects as a result of the modulation (control) of the central nervous system. Specifically, deep pressure touch influences reticular formation activity and autonomic nervous system function [20]. Authors postulate that deep pressure touch provided via weighted blankets offers a feeling of safety, comfort, and groundedness [12]. In some cases, weighted blankets are used to help individuals stabilize and modulate responsiveness to sensory input in order to lower anxiety [12,17,18], level of arousal, decrease impulsivity, increase attention to task, and decrease maladaptive internalizing emotions [12,13].

Sensory integration theory also accounts for varying types of sensory responsivity. Schaaf and Anzalone [24] describe sensory responsivity as the ability to receive, organize, and interpret sensory stimuli across multiple sensory domains/systems including oral, visual, tactile, vestibular, proprioceptive, auditory, and interoception. Therefore, sensory responsivity is “the ability to regulate the response to sensory input” ([23], p. 277). Sensory over-responsivity (SOR) is a subtype of sensory processing disorder where the child or individual responds to a cluster of sensations in an extreme or exaggerated manner [23]. Reynolds, Lane, and Mullen [9] found that children with autism and SOR had more difficulties with sleep than children with only autism. Shochat, Tzischinsky, and Engel-Yeger [22] and Vasak, Williamson, Garden, and Zwiker [21] hypothesized that increased sleep disturbances might be associated with increased sensory sensitivity due to a low neurological threshold and use of a passive self-regulation strategy. Vasak and colleagues [21] also reported that infants and toddlers demonstrating increased sensory sensitivity required a longer time to settle to fall sleep. Evidence exists that links patterns of similar sensory sensitivities with restless behavior and difficulty falling asleep among typical school-aged children and adults [22].

Studies of weighted blanket interventions for children with autism are emerging in the literature. Gringras and colleagues [14] conducted a study with 73 children ages 5–16 with autism who had a concomitant report of a caregiver’s sleep disturbance in the previous five months. The authors implemented a crossover design toggling weighted blanket application for two weeks with a non-weighted blanket. The primary outcome was total sleep time as measured by an actigraph (a wearable device like a watch that continuously measures sleep parameters). Gringras and colleagues’ [14] primary finding for children with a wide range of autism severity levels were that weighted blankets were not any more effective than a typical blanket in helping children with autism improve their total sleep quantity.

Despite the lack of significant findings related to weighted blankets improving sleep quality among children with autism, Gringras and colleagues [14] reported that parent’s/participant’s experienced an improvement in next-day behaviors captured using a sleep diary kept by the participants’ parents/caregivers. Gringras and colleagues [14] hypothesized that an improvement in next day behaviors might have been due to improved bedtime behaviors (i.e., routines). Research design aspects that may have improved overall parent/child interactions include parents wishing to please the study team, or parents observed improvements that the objective measures were not sensitive enough to capture.

Gee and colleagues [15,16] implemented a weighted blanket intervention using a single-subject ABA design in two separate studies. They found minimal changes in sleep duration and morning mood via caregiver report. Gee and colleagues [15,16] examined whether weighted blankets have positively impacted time to fall asleep, the number of wakings, duration of sleep, and morning mood for two children with autism and SOR. Using visual analysis of caregivers’ perceptions, the overall findings demonstrated minimal improvement of the measured constructs related to sleep quality. Participants exhibited evidence of an increase in the total amount of sleep per night and a slight decrease in time to fall asleep. However, morning mood did not consistently improve with the weighted blanket’s use across all participants [15,16].

Finally, a systematic review [25] was conducted evaluating general effectiveness of weighted blankets across various population conditions. The authors concluded that weighted blankets might be an appropriate therapeutic tool in reducing anxiety; however, the authors indicated that more evidence is needed to recommend their use in improving sleep quality among diverse populations.

A paucity of research exists exploring the efficacy of weighted blanket interventions with younger children with autism (e.g., three to six years old), SOR to tactile and auditory stimulus, and sleep disturbances. Therefore, the present study’s primary aim was to examine weighted blankets in younger children with autism, SOR (tactile and auditory sensory domains), and sleep disturbances (difficulty falling asleep, staying asleep, and poor morning mood). A secondary aim was to use intervention and measurement tools commonly utilized in occupational therapy practice and affordable to clinical professionals (e.g., weighted blankets and Sense Sleep App) and caregivers (e.g., weighted blanket).

### Research Question

Does a weighted blanket impact sleep quality among children with autism, sleep disturbances, and sensory over-responsivity?

## 2. Materials and Methods

The current study implemented an ABA research design with pre- and post-test phases [26]. The research design was selected based upon the alignment of the purpose and research question guiding the study. Further, the design is used due to the low availability of a clinical sample in a largely rural and medically underserved area in the Intermountain West region of the United States. The overall study aimed at increasing the duration of the intervention phase, using a caregiver questionnaire tracking participant behavioral changes and using a sample of younger children that were implemented in the Gringras et al. [14] and Gee et al. [15,16] studies.

The pre-test phase consisted of participants’ caregivers completing subjective measures related to their child’s sleep behavior patterns and sensory processing preferences/challenges. The Sensory Processing Measure–Preschool version (SPM-P) [27] and Children’s Sleep Habits Questionnaire (CSHQ) [28] were administered to ensure the participant met the study’s inclusion criteria. The SPM-P is a judgment-based rating scale to measure distinct sensory processing patterns (tactile, vestibular, auditory, visual, etc.), praxis, and social participation among preschool-aged children (3–5 years of age). The CSHQ is a judgment-based rating scale completed by caregivers to measure sleep habits in children ages 4 to 10. The measure has an internal consistency of 0.78 with a sensitivity of 0.80. The classification accuracy of sleep disorders among the targeted age range is 80% [27].

The first phase of the study labeled the baseline phase, lasted for at least seven days. During the baseline phase, the participants’ caregivers completed a five-question, non-standardized Daily Caregiver Survey that quantitatively identified the time to fall asleep at night, duration of night sleep, number of times the child woke up during the night, and a child’s morning mood. The survey was developed as an attempt to integrate recommendations from the Gringras et al. study [14]. After completing the baseline measures, the participants transitioned to a 14-day weighted blanket intervention phase. Throughout the intervention phase, participants slept with a weighted blanket, and the caregivers continued to complete the daily surveys. After completing the intervention phase, the weighted blankets were withdrawn, and the study transitioned into the withdrawal phase. During the withdrawal phase, caregivers continued to complete daily surveys for eight days.

### 2.1. Method of Recruitment

The Human Subjects Committee approved the study at Idaho State University (Pocatello, ID) (on 10 February 2017, IRB-FY2016-170). Study participants were recruited via brochures distributed by the first author and primary investigator (PI) to local pediatricians, pediatric occupational therapists, and speech-language pathologists. Interested caregivers contacted the PI directly to receive additional study details and ask questions. During the initial phone conversation, the PI asked several questions to determine eligibility (see inclusion criteria). If the participant met the inclusion criteria and demonstrated a willingness to participate in the study, written informed consent was obtained. Informed consent was obtained before the participants beginning the study.

### 2.2. Inclusion Criteria

Study participants were required to meet the following inclusion criteria to participate in this study. The child needed to:

(1) have a medical diagnosis (provided by the caregiver and treating physician) of autism;
(2) demonstrate the behavioral manifestations of sensory over-responsivity (*T*-score of 70 or higher on the tactile and/or auditory domains on the SPM-P) [27];
(3) qualitative ratings of “usually” (5 days per week) or higher in multiple aspects of sleep quality on the CSHQ [28];
(4) be between the ages of three and six.

The caregiver needed to:

(1) be able to report if the child had difficulty falling asleep and staying asleep,
(2) speak and understand the English language;
(3) have daily access to a reliable internet connection during the study period;
(4) be able to complete an online Daily Caregiver Survey for 30 days;
(5) be able to implement a weighted blanket as part of the child’s sleep routine for 14 consecutive days.

Participants and caregivers were excluded from the study if they did not meet the above-listed inclusion criteria.

#### Description of the Participants

Participant one, using the pseudonym John, was a four-year, five-month-old male child with a reported autism diagnosis that included a cognitive impairment. The findings from the SPM-P [27] caregiver report screener indicated a Definite Dysfunction in the behavioral manifestations of over-responsivity to tactile (*T*-Score of 72), auditory (*T*-Score of 78), and visual sensory (*T*-Score of 70) stimuli. The qualitative results from the CSHQ [28] caregiver report ratings indicated he demonstrated poor sleep quality as evidenced by difficulty falling asleep (“always”—seven days a week), staying asleep (“always”—seven days a week), wakes up too early (“usually”—five days a week) and experiences a poor morning mood (“usually”—five days a week). No other medical comorbidities were reported.

Participant two using the pseudonym Katie, was a four year, one-month-old female child with a reported diagnosis of autism. The findings from the SPM-P [28] caregiver report screener indicated a Definite Dysfunction in the behavioral manifestations of over-responsivity to tactile (*T*-Score of 80), auditory (*T*-Score of 74), and visual sensory (*T*-Score of 73) stimuli. The qualitative results from the CSHQ caregiver report ratings indicated that she demonstrated difficulty staying asleep (wakes more than once at night (“usually”—five days a week)), wakes up too early (“always”—seven days a week) and experiences a poor morning mood (“usually”—five days a week). No other medical comorbidities were reported.

### 2.3. Dependent Variables

Daily Caregiver Surveys (delivered online via SurveyMonkey^®^) were completed throughout all study phases. The non-standardized survey consisted of six subjective questions assessing the participants’ sleep habits from the previous day and mood the morning the survey was completed. Each survey was completed by the caregiver based upon their best recollection of the previous night’s events. The survey tracked the caregivers’ perception of their child’s sleep latency, number of naps, duration of naps, number of night wakings, sleep duration, and morning mood. Morning mood was operationalized as feelings, varying in intensity and duration, and usually involving more than one emotion [29]. In this case, the authors identified agitation/calm as one emotion related to mood. The assessment of morning mood (i.e., agitation/calm) allowed for the participants’ caregivers to rate the current level of the child’s agitation compared to the prior day using a five-point Likert like scale (more agitated, slightly more agitated, no difference, slightly calmer, and more calm).

The Sense [30] Sleep App was used to objectively track variables, including the participant’s overall sleep quality, total hours of sleep, and the number of hours of deep sleep. The Sense Sleep App included a motion tracker called a “pill” attached to the participants’ pillowcase or sheet at the head of the bed. The tracker’s base component sat next to the bed and captured movement-related information from the pill attached to the participant’s pillow or sheet. The Sense Sleep App exported data that were transmitted and stored from the pill and the base each morning to an Apple Inc. device (e.g., iPad provided by the PI to each participant). Upon return of the iPad at the end of the research study, the data were transferred to a Microsoft Excel spreadsheet. This commercially purchased device had not been utilized in any peer-reviewed literature. Due to the proprietary nature of the device, information related to reliability and validity were unavailable. From a pragmatic perspective, the Sense Sleep App was used because it was a non-wearable system. The target population is young children (3–6 years old) who also demonstrated tactile sensory over-responsivity, which removed the option of using wearable devices such as the Garmin Jr. HR. Additionally, the cost was approximately USD 99.00, which is affordable for most clinicians to utilize in practice.

### 2.4. Intervention

During the intervention phase of the study, participants used weighted blankets for 14 consecutive nights. These weighted blankets were the SensaCalm^®^ brand, custom made, and were provided by the PI. The weighted blankets were designed to be 10% of each child’s body weight adhering to the prototypical weighted blanket protocol [13,14,15,16]. The SensaCalm^®^ blankets used for the study ranged from 3–7 pounds to accommodate the varying weights of potential participants and ranged between USD 40.00 and 80.00. The blanket brand was chosen based upon the affordable cost, durability, equal distribution of weight across the blanket. When the weighted blankets were provided, caregivers were given instructions on safely and effectively using them. Caregivers were instructed to only use the blankets at night (i.e., not during nap time or quiet time); only use the blanket if the child was able to remove it on their own; cover the child’s body, arms, and feet but not their head or face; check on the child occasionally while using the blanket; adjust other bedding while using the weighted blanket to ensure the child was not too hot, and to contact the PI if the weighted blanket was showing signs of wear. Additionally, caregivers of the participants reported that they slept in their own bed (as opposed to the caregivers, or another location) through this study’s duration.

### 2.5. Method of Analysis

Data were analyzed through visual analysis of repeated measure graphs generated using Microsoft Excel, version 16, as described by Kennedy [31]. Visual analysis is widely accepted as a mechanism to analyze data for single-subject designs [32]. The literature supports visual inspection as the preferred method of analysis among single-subject designs because it is sensitive and able to capture intervention effects significant to clinicians working outside research labs within clients’ natural or typical context [32]. Moreover, the visual analysis approach is preferred because it has lower error rates and is conservative enough to identify reliable treatment effects [32].

In addition to visual analysis, this study used the percentage of non-overlapping data (PND) [33] as an additional analysis tool. PND is a statistical method widely used in behavioral science research, particularly for analyzing the small data sets, which are commonplace with single-subject design studies. PND is calculated by identifying the most extreme data point in the baseline phase (either the highest or lowest value depending on whether the intervention is intended to reduce or increase a behavior). The PND is the percentage of data in the intervention phase, which falls above or below this point based on its intended outcome.

## 3. Results

### 3.1. Visual Analysis

**Figure 1:**
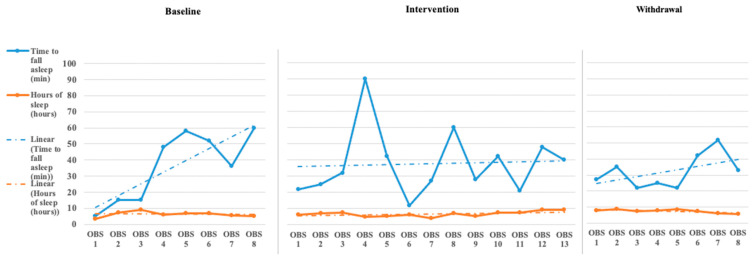
John’s caregiver reported sleep onset latency and sleep duration [reported sleep onset latency is measured 0–60 min and sleep duration is measured 0–10 h (OBS = Observation)].

**Figure 2:**
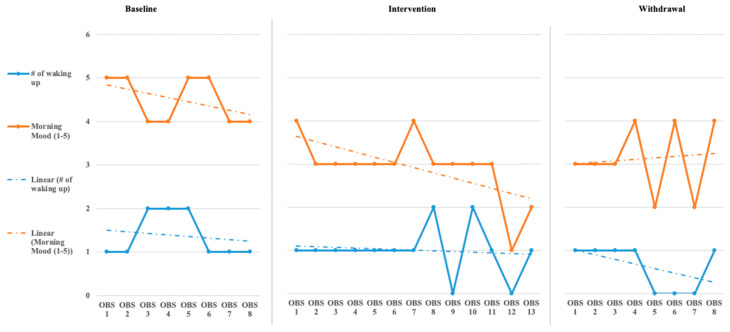
John’s caregiver reported number of wakings and morning mood [reported number of wakings is measured 1–5 times and morning mood ratings are 1–5(OBS = Observation)].

**Figure 3:**
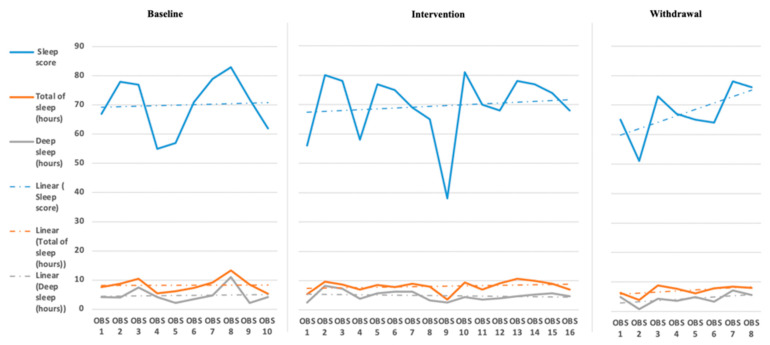
John’s Sleep Sense sleep score sleep duration and deep sleep duration [sleep score is measured between 1 and 10, total sleep hours are measured in 0–12 h, and deep sleep is measured in 0–8 h (OBS = Observation)].

**Figure 4:**
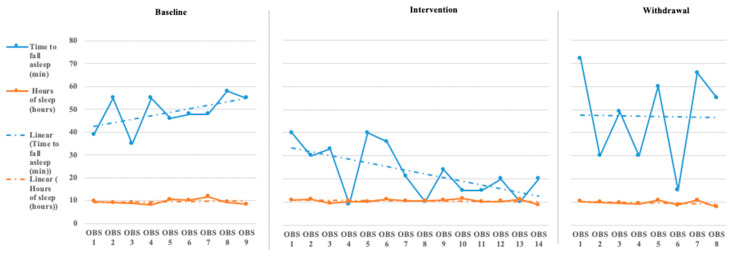
Katie’s caregiver reported sleep onset latency and sleep duration [reported sleep onset latency is measured in minutes and sleep duration is measured in hours (OBS = Observation)].

**Figure 5:**
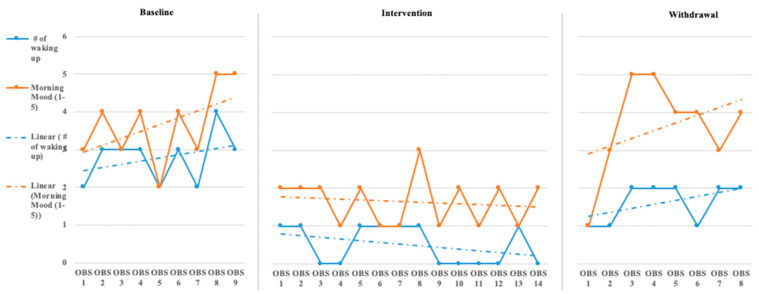
Katie’s caregiver reported number of wakings and morning mood [reported number of wakings is measured 1–5 times and morning mood ratings are 1–5 (OBS = Observation)].

**Figure 6:**
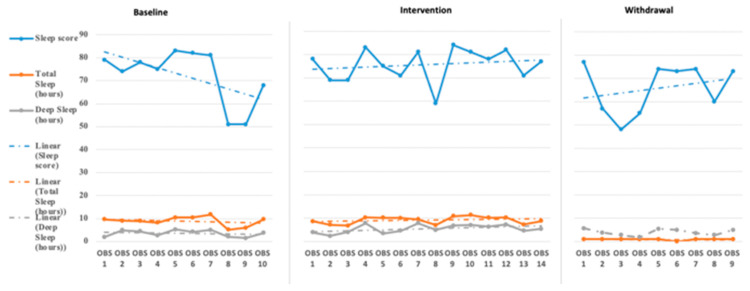
Katie’s Sleep Sense sleep score sleep duration and deep sleep duration (sleep score is measured between 1 and 10, total sleep hours are measured in 0–12 h, and deep sleep is measured in 0–8 h).

The initial step for data analysis for this study was a visual analysis of the data plotted as a figure composed of the scores/ratings from the outcome measures (Daily Caregiver Survey and the Sense Sleep App). The data were evaluated observing changes in level, slope, and variability in data points across each phase for both participants’ subjective and objective measures. Figure 1, Figure 2, Figure 3, Figure 4, Figure 5 and Figure 6 represent the caregiver survey (sleep onset latency, sleep duration, number of night wakings, and morning mood) and the data from the Sleep sense app (sleep score, sleep duration, and deep sleep).

John’s caregiver reported a time to fall asleep during the baseline phase but was reduced to just below 40 min during the intervention and withdrawal phases (see Figure 1). The caregiver’s perception of John’s total hours of sleep did not see a significant change in level or slope across the phases of the study (see Figure 1). The number of night waking times reported by the demonstrated a downward trend across the baseline and intervention phases and leveled off during the withdrawal phases (see Figure 2). Finally, the caregiver’s perception of John’s morning mood began relatively level across the baseline and intervention phases but became improved towards the end of the intervention and withdrawal phases (see Figure 2). Visual analysis of the Sleep sense app for John yielded limited changes in slope, level, or variability (see Figure 3).

Analysis of Katie’s data through visual analysis yielded notable changes in the caregiver’s reporting of time to fall asleep (see Figure 4) during the intervention phase and return to higher levels of approximately 50 min in the withdrawal phase. John’s caregiver noted no significant changes in the total hours of John’s sleep across all phases. Katie’s caregiver reported a slight increase over the baseline phase in the number of night wakings but a small drop and between 1 and 2 times across the intervention phase, returning to higher levels during the withdrawal phase (see Figure 5). Evaluating Katie’s caregiver’s perception of Katie’s morning mood saw a trend on poorer ratings during the baseline phase with a significant improvement in morning mood ratings during the intervention phase with a return to poorer behavioral ratings during the withdrawal phase. Visual analysis of the Sleep sense app for Katie also yielded little changes in slope, level, and variability (see Figure 6).

### 3.2. Quantitative Analysis

The percentage of non-overlapping data (PND) statistic was calculated to assess treatment effectiveness. Calculations were conducted using Microsoft Excel. Scruggs and Mastropieri [33] provide evaluative criteria for implementing this frequently used analysis method for single-case research. The index of treatment effectiveness is based on the percentage of non-overlapping data using the following criteria: PND ≥ 90% = Very Effective, PND 70–90% = Effective, PND 50–70% = Questionable effectiveness, and PND < 50% = Ineffective. When applying these methods in the current study, the only factor categorized as Effective was for John the morning mood category for Katie the categories time to fall asleep, and the number of night wakings (see Table 1 and Table 2).

## 4. Discussion

The primary aim of this study was to assess the weighted blanket application during sleep for young children with autism with sleep difficulties and tactile and auditory behavioral manifestations of SOR. Does a weighted blanket impact sleep quality among children with autism, sleep disturbances and sensory over responsivity? The findings from the two participants indicate that a weighted blanket had little influence with improving sleep quality through the objective and subjective measures. The findings are consistent with the findings of Gringras et al. [14], Gee et al. [15], Eron et al. [25], and Gee et al. [16]. The existing literature generally does not support that weighted blankets improve sleep quality in children with autism. Occupational therapy professionals working with children with autism, SOR, and sleep disturbances have other therapeutic resources to support improved sleep quality.

### 4.1. Limitations

The participants were obtained through convenience sampling methods and were comprised of caregiver–child dyads who volunteered to participate in the study via recruitment brochures. Given that the findings are minor and come from a small sample, a generalization of these results cannot be made.

The application of self-report measurement tools created some challenges. The Daily Caregiver Survey lacked any psychometric analysis; however, a critical component of the current study was to provide caregivers an opportunity to offer sleep quality perceptions and rate their child’s mood throughout the study. Though the survey ratings offered a caregiver-friendly approach, there are inconsistencies in how the caregivers evaluated each participant’s sleep habits, particularly as the caregivers could not be blinded to the study phases.

Culturally, education and healthcare professionals prescribe weighted blankets and vests at 10% of the child’s body weight, though there is no empirical data to support such a practice. For this study, it was difficult to determine if the weight of the blankets used were too light or too heavy.

The Hello Sense Sleep App was a proprietary tool, and, unfortunately, the researchers were not provided with its validity and reliability properties despite multiple requests. The authors did not conduct a thorough reliability study prior to implementing the study. The tracking device was typically attached to the participant’s pillow, yet if the participant left his/her bed to co-sleep with their parent, removing or dislodging of the tracking device from the pillow or sheet, a gap in the data collection would be introduced.

This highlights a challenge between finding an objective measure that can track sleep outcomes but not cause additional difficulty in tactile SOR that could be caused by a wearable sleep tracking device. This study should be replicated with different and potentially more reliable wearable sleep tracking devices appropriate for pediatric populations with tactile SOR.

Finally, the study’s duration, 30 days with 14 days of intervention, may not have been long enough to measure a functional change. The rate at which the participants habituate to having a new/weighted blanket may have been slower than what the study could have captured.

### 4.2. Recommendations for Future Research

Future research could employ a mixed-methods approach, exploring objective measures either as repeated measures or pre- and post-outcomes related to sleep quality. These outcomes could be paired with caregiver qualitative journals focused on documenting the child’s physical activity during the day, evening rituals related to sleep hygiene, and their child’s mood the morning after the use of a weighted blanket. A mixed-methods approach could capture missing data from the parent/caregiver’s perspective on sleep quality changes while using a weighted blanket with their child. Further research could explore pairing structured sleep hygiene rituals with the child and their parent/caregiver along with a weighted blanket to explore how changes in habits and routines and a sensory-based intervention influence sleep quality in children. More robust research exploring the effectiveness of weighted blankets on improving sleep quality among children with autism is needed. Specifically, research studies using a control (or waitlist) group, larger sample sizes in both the control and experimental group. Further research studies should employ reliable yet affordable objective sleep-related measurement devices that would support the tactile SOR related challenges among some children with autism.

### 4.3. Key Points for Clinicians

- Weighted blankets for the use of improved sleep quality for children with autism continues to be experimental.
- Clinical professionals using weighted blankets with children with autism should pay close attention to the underlying factors contributing to the child’s sleep disturbances (behavioral, biological, environmental, sensory, culture, etc.).
- Clinical professionals need to establish a sound clinical hypothesis for using a weighted blanket with children with autism; monitoring the response to the intervention over time will help support the plan of care on the functional implications of the intervention.
- Clinical professionals should collaborate with the child with autism, medical doctor, and caregiver to identify the underlying mechanisms. Based upon the findings from this and other studies, the use of a weighted blanket to enhance sleep quality in children should be used judiciously; the evidence from this study and other studies indicate that there are more effective approaches to improving sleep quality in children with autism.

## Figures and Tables

**Table 1:** John and Katie percentage of non-overlapping data (PND) analysis of daily caregiver survey.

Daily Caregiver Survey			
A1 Phase—Baseline	PND Baseline—Low	PND Baseline—High	PND Selected
John time to fall asleep (min)	6	60	Low
John Sleep Duration	3.5	9.2	High
John Number of Night Wakings	1	2	Low
John Morning Mood	4	5	Low
Katie Time to Fall Asleep (min)	35	55	Low
Katie Sleep Duration	8.5	11.8	High
Katie Number of Night Wakings	2	4	Low
Katie Morning Mood	2	5	Low
B Phase—Intervention	Number of days PND	PND% out of 14 or 7 days	PND Interpretation
John time to fall asleep (min)	1	0.07	Ineffective
John Sleep Duration	0	0.00	Ineffective
John Number of Night Wakings	2	0.15	Ineffective
John Morning Mood	11	0.84	Effective
Katie Time to Fall Asleep (min)	11	0.84	Effective
Katie Sleep Duration	0	0.00	Ineffective
Katie Number of Night Wakings	13	1.00	Effective
Katie Morning Mood	6	0.46	Ineffective

PND ≥ 90% = Very Effective, PND 70–90% = Effective, PND 50–70% = Questionable Effectiveness, and PND < 50% = Ineffective.

**Table 2:** John and Katie PND analysis Sense Sleep App.

Sleep Sense App Analysis			
A1 Phase—Baseline Testing	PND Baseline—Low	PND Baseline—High	PND Selected
John Sleep Score	55	83	High
John Sleep Duration	5.3	11.8	High
John Deep Sleep Duration	2.1	11	High
Katie Sleep Score	51	83	High
Katie Sleep Duration	5.1	11.8	High
Katie Deep Sleep Duration	1.5	5.3	High
B Phase—Intervention	Number of days PND	PND% out of 14 or 7 days	PND Interpretation
John Sleep Score	1	0.06	Ineffective
John Sleep Duration	1	0.06	Ineffective
John Deep Sleep Duration	0	0.00	Ineffective
Katie Sleep Score	1	0.06	Ineffective
Katie Sleep Duration	0	0.00	Ineffective
Katie Deep Sleep Duration	7	0.46	Ineffective

PND ≥ 90% = Very Effective, PND 70–90% = Effective, PND 50–70% = Questionable Effectiveness, and PND < 50% = Ineffective.

## Data Availability

The data presented in this study are available on request from the corresponding author. The data are not publicly available due to being stored for only five years in accordance with human subject approval.
